# Supplementary material for: Atypical heat shock transcription factor HSF5 is critical for male meiotic prophase under non-stress conditions
Source: Nat Commun. 2024 Apr 29;15:3330. doi: 10.1038/s41467-024-47601-0 (PMC11059408; doi:10.1038/s41467-024-47601-0)
Supplement: Supplementary file 1 — Supplementary Information [file 41467_2024_47601_MOESM1_ESM.pdf]

Supplementary information

Atypical heat shock transcription factor HSF5 is critical for male meiotic prophase under non-stress conditions

Saori Yoshimura[1,2,7], Ryuki Shimada[1,7], Koji Kikuchi[1], Soichiro Kawagoe[3], Hironori Abe[1], Sakie Iisaka[1], Sayoko Fujimura[4], Kei-ichiro Yasunaga[4], Shingo Usuki[4], Naoki Tani[4], Takashi Ohba[2], Eiji Kondoh[2], Tomohide Saio[3], Kimi Araki[5, 6], and Kei-ichiro Ishiguro[1*]

Affiliations:
[1] Department of Chromosome Biology, Institute of Molecular Embryology and Genetics (IMEG), Kumamoto University, Honjo 2-2-1, Chuo-ku, Kumamoto, 860-0811, Japan
[2] Department of Obstetrics and Gynecology, Faculty of Life Sciences, Kumamoto University, 860-8556 Japan
[3] Division of Molecular Life Science, Institute of Advanced Medical Sciences, Tokushima University, Tokushima 770-8503, Japan.
[4] Liaison Laboratory Research Promotion Center, IMEG, Kumamoto University, Kumamoto 860-0811, Japan
[5] Institute of Resource Development and Analysis, Kumamoto University, Kumamoto, 860-0811 Japan
[6] Center for Metabolic Regulation of Healthy Aging, Kumamoto University, Kumamoto 860-8556, Japan.
[7] These authors equally contributed to this study : Saori Yoshimura, Ryuki Shimada
*Correspondence to: Kei-ichiro Ishiguro, Ph.D. Email: ishiguro@kumamoto-u.ac.jp

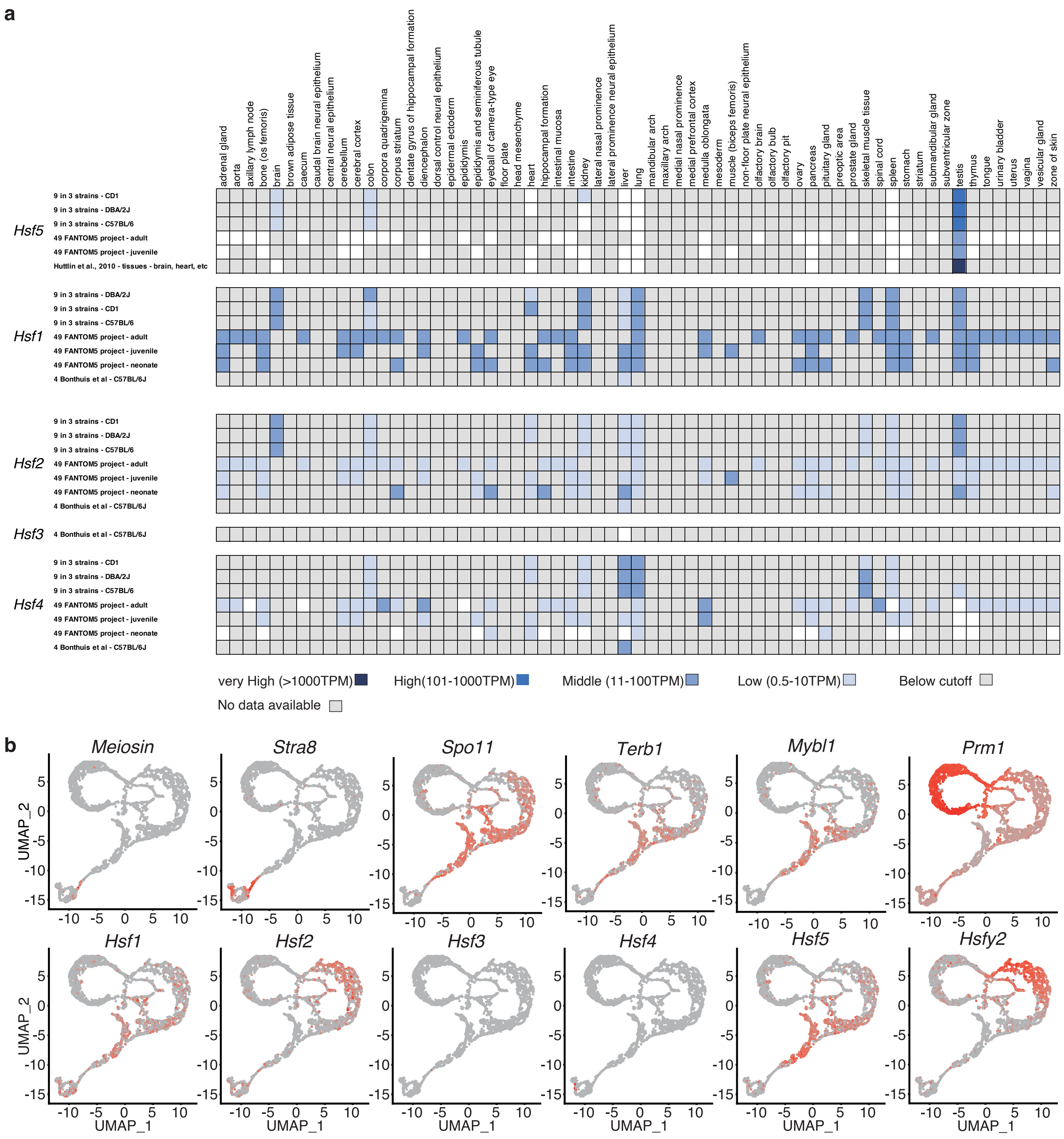

Supplementary Figure 1. Specific expression of Hsf5 orthologs in mouse and human testis.

(a) The tissue expression atlas of mouse Hsf gene paralogs are adapted from Expression Atlas (https://www.ebi.ac.uk/gxa/home). The expression levels (TPM : transcripts per million) are shown with the indicated color codes.

(b) UMAP plots show expression patterns of mouse Hsf gene paralogs and other key developmental genes are reanalyzed using public scRNA-seq data of spermatogenic cells in adult mouse testis (GEO: GSE109033) (Hermann et al. 2018). Key developmental marker genes include *Stra8*: differentiating spermatogonia and pleleptotene spermatocyte, *Meiosin*: pleleptotene spermatocyte, *Spo11*, *Terb1*: meiotic prophase spermatocyte, *Mybl1*: pachytene spermatocyte, *Prm1*: round and elongated spermatid.

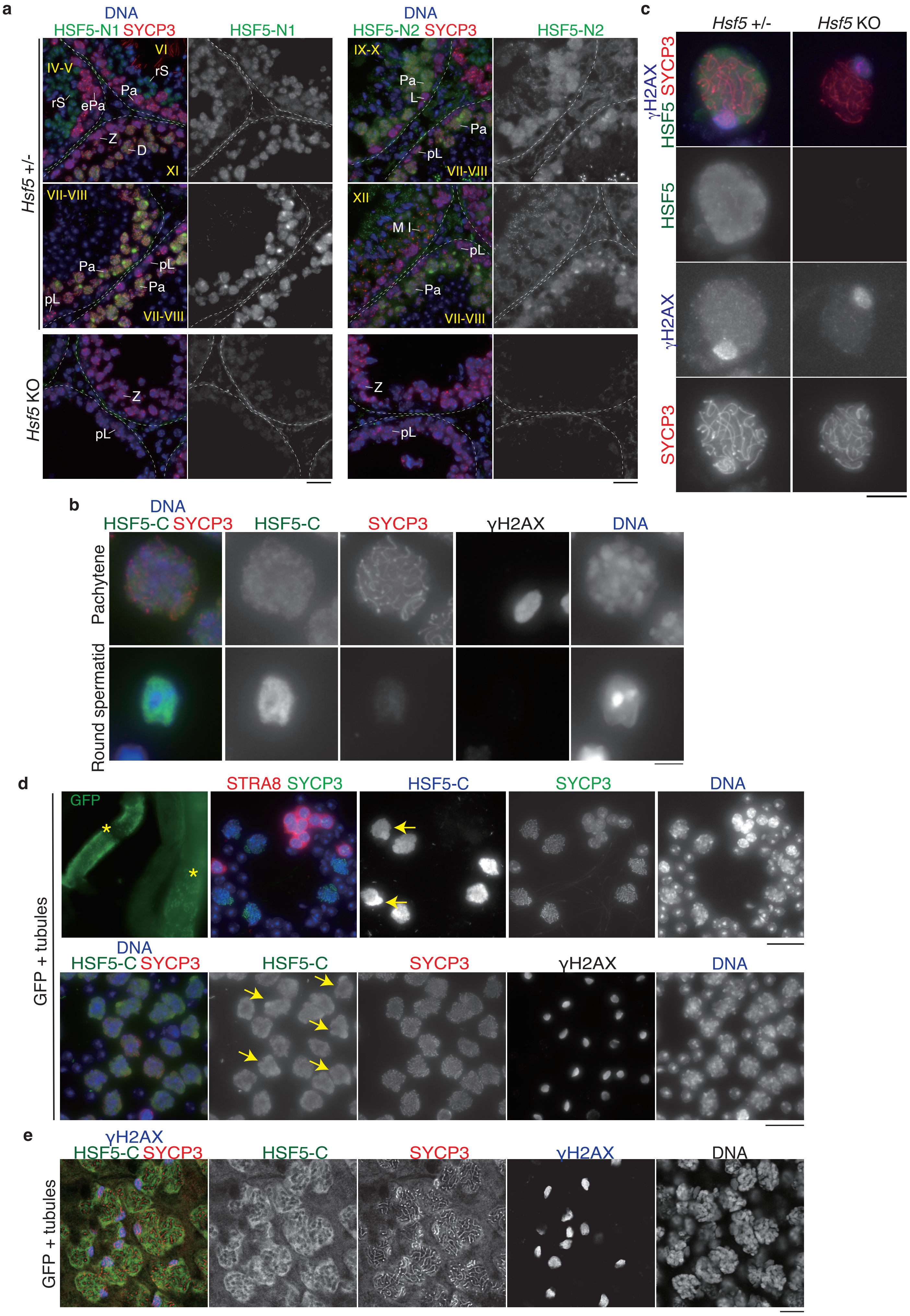
a
DNA
HSF5-N1 SYCP3
HSF5-N1
DNA
HSF5-N2 SYCP3
HSF5-N2
Hsf5 +/-
Hsf5 KO
VI
IV-V
rS
Pa
ePa
Z
D
XI
VII-VIII
pL
IX-X
L
XII
M I
c
Hsf5 +/-
Hsf5 KO
γH2AX
HSF5
SYCP3
b
Pachytene
Round spermatid
HSF5-C
DNA
d
GFP + tubules
GFP
STRA8 SYCP3
e

Supplementary Figure 2. Immunostaining of HSF5 in the mouse spermatocytes

(a) Seminiferous tubule sections in WT testis (8-weeks old) were immunostained as indicated. The same immunostaining patterns of HSF5 were confirmed by HSF5-N1 and HSF5-N2 antibodies. pL: preleptotene, L: Leptotene, Z: Zygotene, ePa: early Pachytene, P: Pachytene, M I: Metaphase I, rS: round Spermatid. Boundaries of the seminiferous tubules are indicated by white dashed lines. Roman numbers indicate the seminiferous tubule stages. Scale bar: 25 μm.

(b) Squashed pachytene spermatocytes and round spermatids were immunostained as indicated. Scale bar: 5 μm.

(c) Pachytene spermatocytes isolated by FACS from *Hsf5* +/- and *Hsf5* KO mice were immunostained as indicated. Scale bar: 10 μm.

(d) The stage VII-VIII seminiferous tubules with GFP fluorescence (*) and GFP negative seminiferous tubule were excised from *Stra8-3xFLAG-HA-p2A-GFP* knock-in mouse (Ishiguro et al. 2020). Pachytene spermatocytes were squashed from the excised seminiferous tubules and were immunostained as indicated. Scale bar: 25 μm. Arrows indicate pachytene spermatocytes with an intense HSF5 signal associated with XY chromosome. A single experiment was performed.

(e) Whole mount immunostaining of the stage VII-VIII seminiferous tubules excised from *Stra8-3xFLAG-HA-p2A-GFP* knock-in mice was assessed by confocal microscopy. Scale bar: 10 μm. Two technically independent experiments from one animal were repeated and showed similar results.

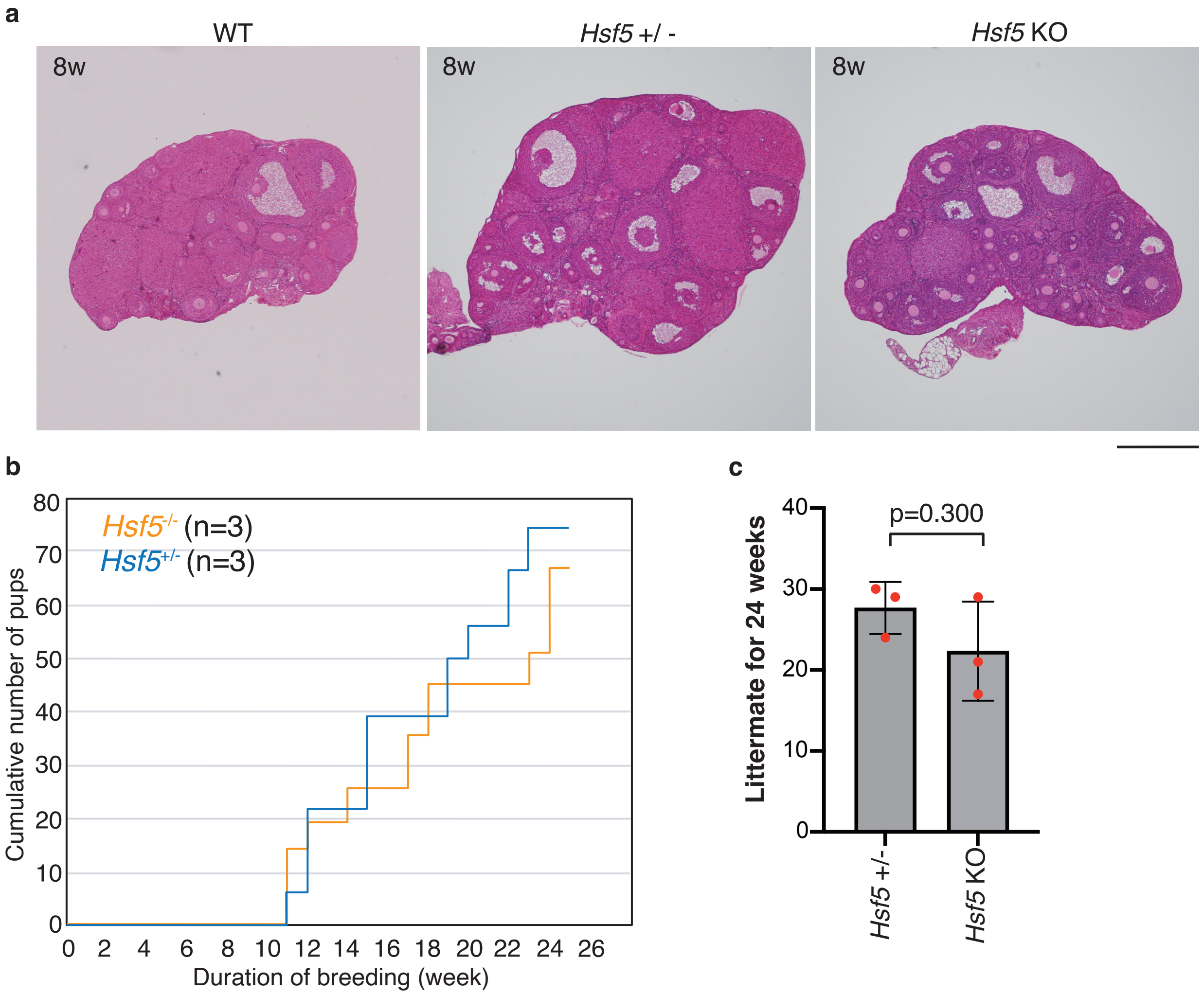

Supplementary Figure 3. Phenotypic analyses of *Hsf5* KO females.
(a) Hematoxylin and Eosin stained sections of WT, *Hsf5*+/- and *Hsf5* KO ovaries (8- weeks old). Biologically independent mice (n=3) for each genotype were examined. Scale bar: 500µm.
(b) Cumulative number of pups born from *Hsf5*+/- (n=3, all 4-weeks old at the start point of mating) and *Hsf5* KO (n=3, all 4-weeks old at the start point of mating) females.
(c) Fertility of *Hsf5* +/- and *Hsf5* KO females was examined by mating with WT males for the indicated period. Litter size is shown on the graph (Mean with SD).

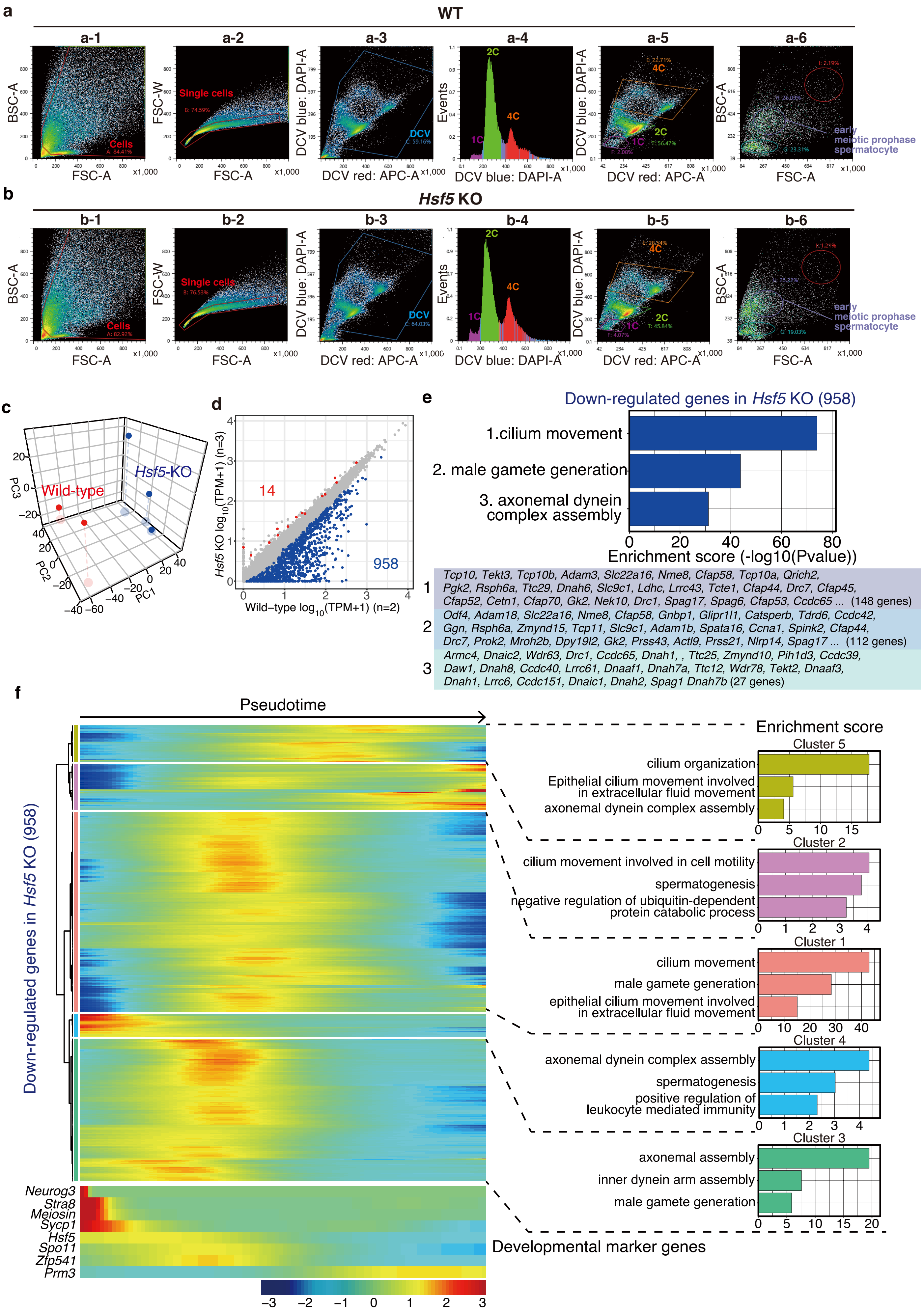
a
WT
a-1
a-2
a-3
a-4
a-5
a-6
BSC-A
FSC-A
x1,000
Cells
FSC-W
Single cells
DCV blue: DAPI-A
DCV red: APC-A
DCV
Events
2C
4C
1C
early
meiotic prophase
spermatocyte
b
Hsf5 KO
b-1
b-2
b-3
b-4
b-5
b-6
c
Wild-type
Hsf5-KO
PC1
PC2
PC3
d
Hsf5 KO log10(TPM+1) (n=3)
Wild−type log10(TPM+1) (n=2)
14
958
e
Down-regulated genes in Hsf5 KO (958)
1.cilium movement
2. male gamete generation
3. axonemal dynein complex assembly
Enrichment score (-log10(Pvalue))
1 Tcp10, Tekt3, Tcp10b, Adam3, Slc22a16, Nme8, Cfap58, Tcp10a, Qrich2, Pgk2, Rsph6a, Ttc29, Dnah6, Slc9c1, Ldhc, Lrrc43, Tcte1, Cfap44, Drc7, Cfap45, Cfap52, Cetn1, Cfap70, Gk2, Nek10, Drc1, Spag17, Spag6, Cfap53, Ccdc65 ... (148 genes)
2 Odf4, Adam18, Slc22a16, Nme8, Cfap58, Gnbp1, Glipr1l1, Catsperb, Tdrd6, Ccdc42, Ggn, Rsph6a, Zmynd15, Tcp11, Slc9c1, Adam1b, Spata16, Ccna1, Spink2, Cfap44, Drc7, Prok2, Mroh2b, Dpy19l2, Gk2, Prss43, Actl9, Prss21, Nlrp14, Spag17 ... (112 genes)
3 Armc4, Dnaic2, Wdr63, Drc1, Ccdc65, Dnah1, , Ttc25, Zmynd10, Pih1d3, Ccdc39, Daw1, Dnah8, Ccdc40, Lrrc61, Dnaaf1, Dnah7a, Ttc12, Wdr78, Tekt2, Dnaaf3, Dnah1, Lrrc6, Ccdc151, Dnaic1, Dnah2, Spag1 Dnah7b (27 genes)
f
Pseudotime
Down-regulated genes in Hsf5 KO (958)
Enrichment score
Cluster 5
cilium organization
Epithelial cilium movement involved in extracellular fluid movement
axonemal dynein complex assembly
Cluster 2
cilium movement involved in cell motility
spermatogenesis
negative regulation of ubiquitin-dependent protein catabolic process
Cluster 1
cilium movement
male gamete generation
epithelial cilium movement involved in extracellular fluid movement
Cluster 4
axonemal dynein complex assembly
spermatogenesis
positive regulation of leukocyte mediated immunity
Cluster 3
axonemal assembly
inner dynein arm assembly
male gamete generation
Neurog3
Stra8
Meiosin
Sycp1
Hsf5
Spo11
Zfp541
Prm3
Developmental marker genes
−3 −2 −1 0 1 2 3

Supplementary Figure 4. SMART-seq analysis of meiotic prophase spermatocytes in *Hsf5* KO

For SMART RNA-seq, meiotic prophase spermatocytes were isolated from **(a)** WT and **(b)** *Hsf5* KO testes at P17 by fluorescent sorting with DCV staining. **(a-1)(a-2)(b-1)(b-2)** Debris and non-single cells were excluded by light scattering. **(a-3)(b-3)** Unstained cell and side population exclusion based on DCV fluorescence. **(a-4)(b-4)** DNA content determination based on DCV-blue fluorescence. **(a-5)(b-5)** Gating on E as a population with DNA content of 4C based on DCV-blue/DCV-red fluorescence. **(a-6)(b-6)** Back-gating of Gate E from the DCV plot on an FSC/BSC plot. We isolated cells gating on H as early meiotic prophase spermatocytes (Yeh et al. 2021). Precise gating of 4C testicular populations was confirmed by SYCP3+ positive immunostaining.

(**c**) The early meiotic prophase spermatocytes (zygotene/pachytene) were isolated from control WT (n=2) and *Hsf5* KO (n=3) testes at P17 by fluorescent sorting, and subjected to SMART RNA-seq. Principal component analysis of the transcriptomes of meiotic prophase spermatocytes in WT and *Hsf5* KO is shown.

(d) Scatter plot of the transcriptome of meiotic prophase spermatocytes in WT versus *Hsf5* KO is shown. The numbers of differentially expressed genes are shown. Significance criteria: false discovery rate ≤ 0.05.

(e) Gene enrichment analysis of the 958 downregulated genes in meiotic prophase spermatocytes of *Hsf5* KO testes. The enriched term for 14 upregulated genes is not shown due to low statistical significance. See Supplementary Data 3 for complete gene list of the Gene enrichment analyses.

(f) Heatmap showing the hierarchical relationship among the clusters of the downregulated genes in *Hsf5* KO across pseudotime of spermatogenesis. The mRNA levels of the downregulated genes in meiotic prophase spermatocytes of *Hsf5* KO was assessed by reanalyzing scRNA-seq data of spermatogenic cells (GEO : GSE109033) (Hermann et al. 2018). Pseudotime (left to right) corresponds to developmental trajectory of spermatogenesis (undifferentiated spermatogonia to round spermatids). For a reference, Expression profiles of key developmental marker genes are shown along pseudotime. The enriched terms (top 3 by enriched score) are shown on the right.

a

	Platform: Chromium (10x Genomics)	
Sampling stage:	**P16**	
Genotype:	wild type	*Hsf5*-KO
Cell type:	whole testicular cells	
Number of used indivisuals:	3	3
Total number of genes detected:	22,518	22,106
Sequencing Saturation:	72.6%	70.3%
Mean reads / cell:	40,968	32,472
Median genes / cell:	1,312	1,278
Median UMI counts / cell:	2,918	2,673
Total number of cells:	9,275	11,023
Number of analyzed testicular germ cells:	1,087 (11.7%)	1,911 (17.3%)

b

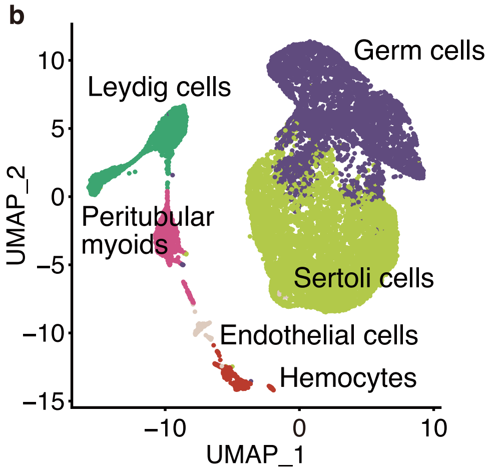

c

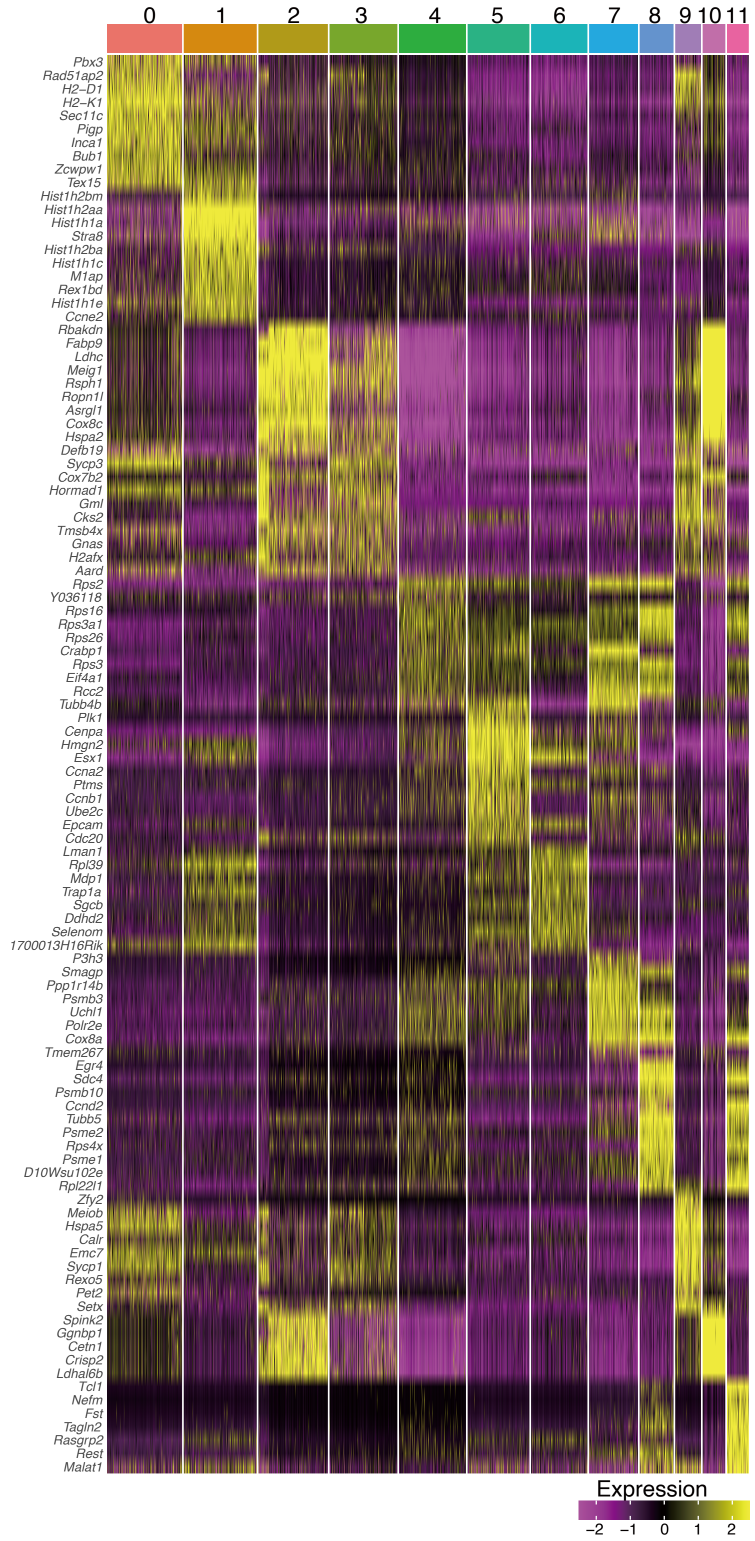

d

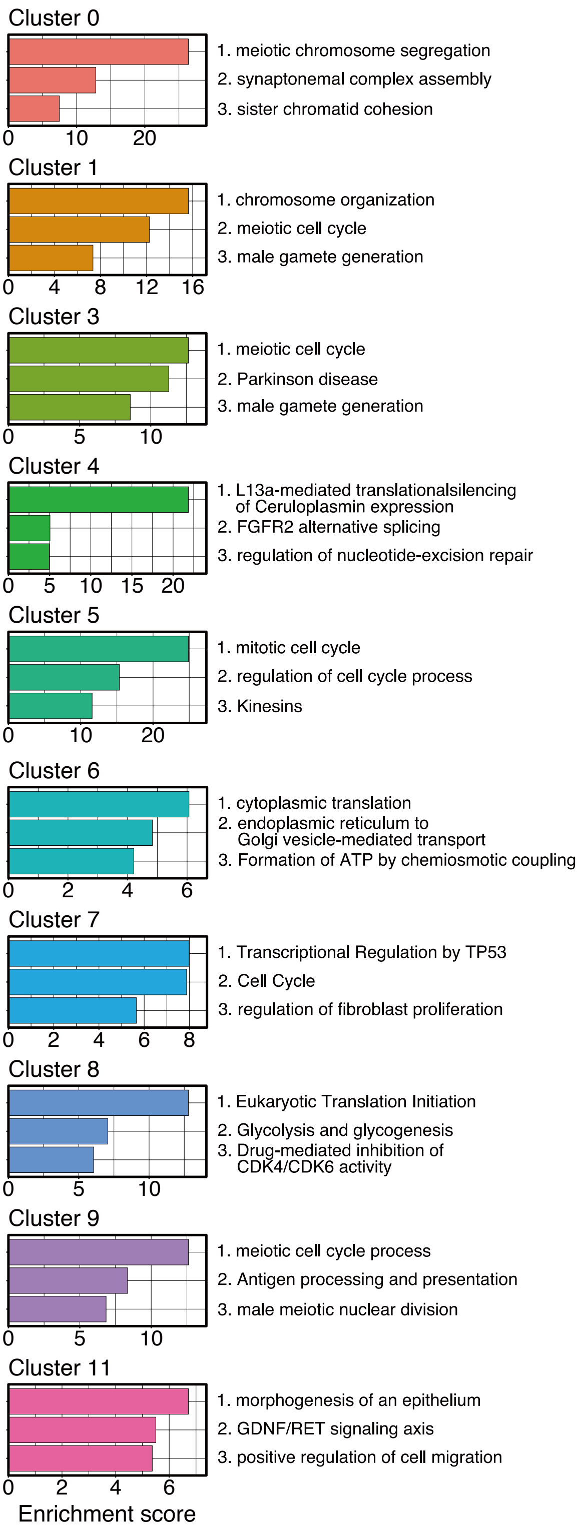

Supplementary Figure 5. Clustering analysis of scRNA-seq data of WT and *Hsf5* KO spermatogenic germ cells.

(a) Summary table of the 10X Genomics Chromium metrics for scRNA-seq analysis with WT and *Hsf5* KO testicular germ cells (P16). Indicated numbers of testes were pooled. Total number of testicular germ cells that were subjected to RNA-seq analysis and separated from other testicular somatic cells are shown. Percentages of the extracted testicular germ cells per total single cells that were subjected to RNA-seq analysis are shown.

(b) UMAP representation of scRNA-seq transcriptome profiles for testicular cells from P16 WT and *Hsf5* KO testes.

(c) Heat plot for the top 10 representative genes of each cluster. The relative expression levels are indicated with the color codes.

(d) Gene enrichment analysis of DEGs in the UMAP-defined cell clusters.

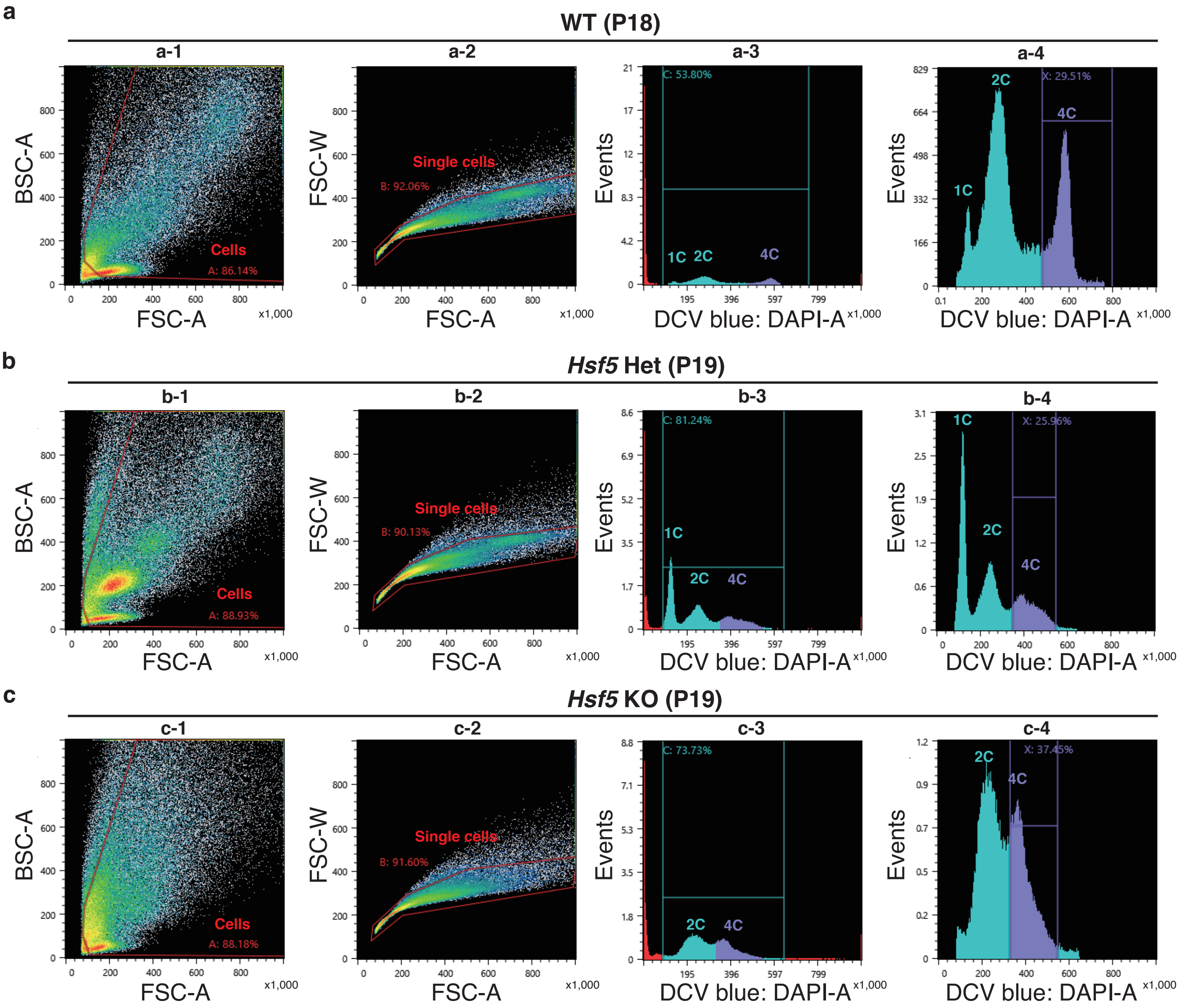

Supplementary Figure 6. Fluorescent sorting of meiotic prophase spermatocytes enriched by DCV and light scattering for CUT&Tag.

For CUT&Tag, meiotic prophase spermatocytes were isolated from **(a)** WT at P18, **(b)** *Hsf5* +/- at P19 and **(c)** *Hsf5* KO testes at P19 by fluorescent sorting with DCV staining. **(a-1)(a-2)(b-1)(b-2)(c-1)(c-2)** Debris and non-single cells were excluded by light scattering. **(a-3)(a-4)(b-3)(b-4)(c-3)(c-4)** DNA content determination based on DCV-blue fluorescence. Gating on X as a population with DNA content of 4C based on DCV-blue fluorescence. We isolated cells gating on X as 4C spermatocytes (Yeh et al. 2021). Precise gating of 4C testicular populations was confirmed by SYCP3+ positive immunostaining.

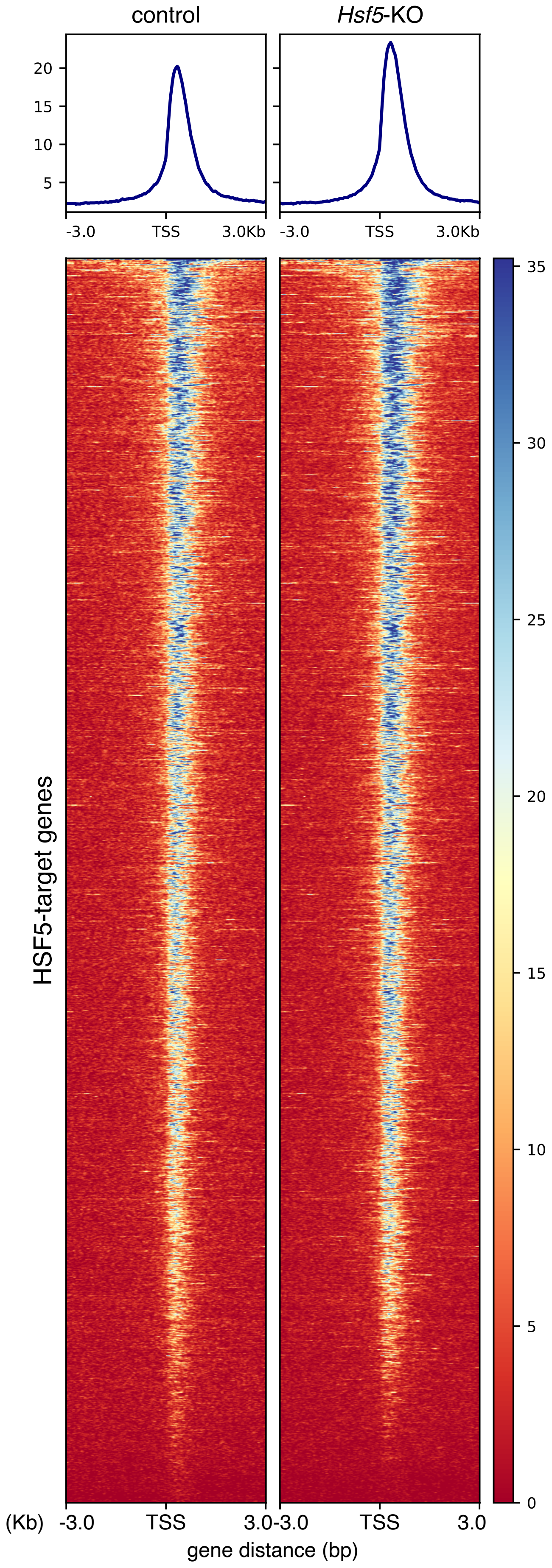

Supplementary Figure 7. ATAC-seq analysis of HSF5-bound genes.
The alterations in chromatin accessibility within pachytene spermatocytes of the control (n=1) and *Hsf5* KO (n=1) were assessed by ATAC-seq. This analysis revealed a modest elevation in chromatin accessibility specifically at transcription start sites (TSSs) among HSF5-target genes in the *Hsf5* KO spermatocytes.

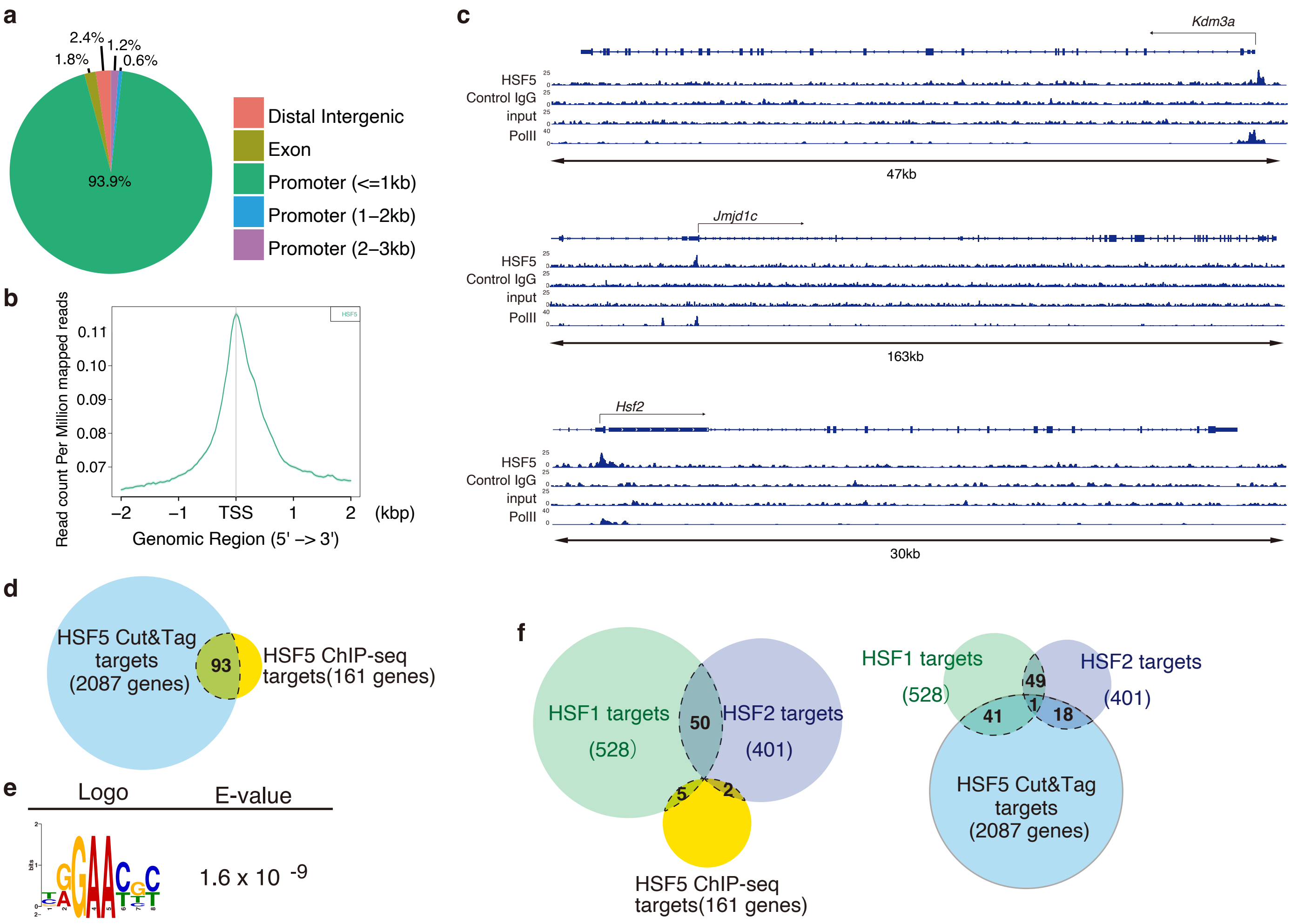

Supplementary Figure 8. ChIP-seq analysis of HSF5 binding sites.

(a) HSF5 binding sites were identified by MACS with option (-g mm -p 0.00001) and classified by the genomic locations as indicated using ChIPSeeker.

(b) Heat map of the common HSF5 binding sites of HSF5 ChIP-seq at the positions -2.0 kb upstream to +2.0 kb downstream relative to the TSS. Average distributions of HSF5- ChIP-seq binding peak are shown on the bottom.

(c) Genomic view of HSF5 ChIP-seq control IgG ChIP-seq input DNA data over representative gene loci. Genomic coordinates were obtained from RefSeq. RefSeq IDs for mRNA isoforms are indicated. To specify testis specific transcription, RNA polymerase II ChIP-seq in the testis are shown (Li et al. 2013).

(d) Venn diagram representing the overlap of between HSF5-bound genes identified ChIP-seq and those by CUT&Tag.

(e) The top sequence motif enriched in HSF5 ChIP-seq with E-values. MEME-ChIP E-value estimates the expected number of motifs with similar features that one would find in a similarly sized set of random sequences. See also Supplementary Data 5.

(f) Venn diagram representing the overlap among the targets of HSF1 (Akerfelt et al. 2010b), HSF2 (Akerfelt et al. 2008), and HSF5 in the testes.

a

Accession	Description	# AAs	HSF5-N1 IP (High salt)			HSF5-N2 IP (High salt)		
			Score	PSMs	Area	Score	PSMs	Area
P17156	Heat shock-related 70 kDa protein 2 [HSP72_MOUSE]	633	573.65	34	4.729E7	1019.86	59	1.081E8
Q9EPU4	Cleavage and polyadenylation specificity factor subunit 1 [CPSF1_MOUSE]	1441	25.54	1	1.067E6	705.52	49	5.726E7
Q9EQK5	Major vault protein [MVP_MOUSE]	861	1265.42	72	5.394E7	622.00	48	2.272E7
Q5ND04	Heat shock factor protein 5 [HSF5_MOUSE]	624	691.95	52	2.569E8	441.77	34	1.812E8
P63017	Heat shock cognate 71 kDa protein [HSP7C_MOUSE]	646	218.90	17	3.756E7	434.93	24	9.073E7
P68372	Tubulin beta-4B chain [TBB4B_MOUSE]	445	316.81	18	3.767E7	388.87	16	5.121E7
Q03265	ATP synthase subunit alpha, mitochondrial [ATPA_MOUSE]	553	297.39	17	1.477E7	366.89	19	1.787E7
P38647	Stress-70 protein, mitochondrial [GRP75_MOUSE]	679	312.83	12	1.249E7	333.10	16	1.745E7
P99024	Tubulin beta-5 chain [TBB5_MOUSE]	444	271.78	14	3.290E7	318.63	14	4.575E7
P20152	Vimentin [VIME_MOUSE]	466	137.88	12	1.698E7	304.21	18	1.879E7
P60710	Actin, cytoplasmic 1 [ACTB_MOUSE]	375	295.67	17	4.417E7	302.22	17	5.940E7
P05214	Tubulin alpha-3 [TBA3_MOUSE]	450	290.31	13	4.465E7	280.25	15	5.056E7
P68369	Tubulin alpha-1A [TBA1A_MOUSE]	451	317.53	13	4.647E7	276.65	15	4.885E7
Q9ERD7	Tubulin beta-3 [TBB3_MOUSE]	450	225.86	12	2.475E7	275.08	14	2.732E7
P08113	Endoplasmin [ENPL_MOUSE]	802	179.22	10	6.059E6	243.51	9	7.597E6
P56480	ATP synthase subunit beta, mitochondrial [ATPB_MOUSE]	529	308.82	16	2.095E7	240.35	16	2.534E7
Q8K310	Matrin-3 [MATR3_MOUSE]	846	121.02	10	6.348E6	238.97	13	1.208E7
Q8VEK3	Heterogeneous nuclear ribonucleoprotein U [HNRPU_MOUSE]	800	119.56	9	1.059E7	222.33	9	1.563E7
Q91YQ5	Dolichyl-diphosphooligosaccharide--protein glycosyltransferase subunit 1 [RPN1_MOUSE]	608	144.81	7	7.731E6	216.28	10	1.286E7
Q9D0E1	Heterogeneous nuclear ribonucleoprotein M [HNRPM_MOUSE]	729	137.64	10	9.755E6	186.96	9	1.766E7
Q61584	Fragile X mental retardation syndrome-related protein 1 [FXR1_MOUSE]	677	139.85	9	1.097E7	127.79	6	6.214E6
Q7TSG5	SH3 domain-containing protein 21 [SH321_MOUSE]	549	121.41	5	7.370E6	120.84	6	8.156E6
Q8CGW4	Transcription factor SOX-30 [SOX30_MOUSE]	782	73.60	7	1.121E7	115.58	6	8.326E6
Q9DA79	Dipeptidase 3 [DPEP3_MOUSE]	493	105.14	8	2.278E7	108.19	7	1.688E7
O54724	Polymerase I and transcript release factor [PTRF_MOUSE]	392	191.19	8	1.823E7	104.32	3	6.814E6
P11499	Heat shock protein HSP 90-beta [HS90B_MOUSE]	724	91.91	2	4.565E6	101.92	2	6.027E6
P29341	Polyadenylate-binding protein 1 [PABP1_MOUSE]	636	51.03	1	4.204E6	98.73	3	3.601E6
P70372	ELAV-like protein 1 [ELAV1_MOUSE]	326	65.26	2	4.341E6	96.54	2	6.476E6
Q9JHU4	Cytoplasmic dynein 1 heavy chain 1 [DYHC1_MOUSE]	4644	62.00	5	2.529E6	91.14	8	3.770E6
O70133	ATP-dependent RNA helicase A [DHX9_MOUSE]	1380	63.95	5	4.121E6	90.18	8	5.170E6
P32037	Solute carrier family 2, facilitated glucose transporter member 3 [GTR3_MOUSE]	493	158.11	6	2.152E7	89.06	5	2.909E7
O54734	Dolichyl-diphosphooligosaccharide--protein glycosyltransferase 48 kDa subunit [OST48_MOUSE]	441	61.91	2	3.362E6	82.39	3	5.284E6
P62806	Histone H4 [H4_MOUSE]	103	45.36	5	5.000E6	78.86	5	4.503E6
P27773	Protein disulfide-isomerase A3 [PDIA3_MOUSE]	505	33.29	2	3.625E6	78.22	4	5.319E6
Q99PU5	Long-chain-fatty-acid--CoA ligase ACSBG1 [ACBG1_MOUSE]	721	29.75	2	2.054E6	78.03	3	2.064E6
Q8CGW9	Doublesex- and mab-3-related transcription factor C2 [DMRTD_MOUSE]	370	70.36	5	9.144E6	75.37	3	3.111E6
Q8BMS1	Trifunctional enzyme subunit alpha, mitochondrial - [ECHA_MOUSE]	763	30.93	2	2.175E6	74.97	3	2.057E6
Q8C2Q3	RNA-binding protein 14 [RBM14_MOUSE]	669	26.55	1	2.314E6	74.90	4	5.640E6
P14733	Lamin-B1 [LMNB1_MOUSE]	588	73.23	7	5.381E6	71.52	4	6.033E6
P61407	Tudor domain-containing protein 6 [TDRD6_MOUSE]	2134	48.13	3	1.540E6	66.69	5	3.593E6
Q8VIJ6	Splicing factor, proline- and glutamine-rich [SFPQ_MOUSE]	699	42.29	3	1.733E6	65.61	3	1.617E6
P52194	Calmegin [CLGN_MOUSE]	611	48.34	2	4.603E6	65.50	3	7.966E6
Q01320	DNA topoisomerase 2-alpha [TOP2A_MOUSE]	1528	126.00	7	7.386E6	64.92	2	5.669E6
Q9DB77	Cytochrome b-c1 complex subunit 2, mitochondrial [QCR2_MOUSE]	453	33.38	1	2.741E6	62.94	2	7.272E6

Accession	Description	# AAs	HSF5-N1 IP (High salt)			HSF5-N2 IP (High salt)		
			Score	PSMs	Area	Score	PSMs	Area
P0CG49	Polyubiquitin-B [UBB_MOUSE]	305	55.68	4	2.408E7	61.72	5	2.220E7
P57776	Elongation factor 1-delta [EF1D_MOUSE]	281	39.17	1	3.438E6	59.96	2	4.674E6
P35564	Calnexin [CALX_MOUSE]	591	32.23	1	2.191E6	59.45	3	1.953E6
O09106	Histone deacetylase 1 [HDAC1_MOUSE]	482	22.51	3	2.412E6	58.75	2	1.911E6
Q61656	Probable ATP-dependent RNA helicase DDX5 [DDX5_MOUSE]	614	28.16	1	3.101E6	57.52	2	3.231E6
Q3V132	ADP/ATP translocase 4 [ADT4_MOUSE]	320	40.21	2	3.137E6	56.24	2	3.792E6
P10126	Elongation factor 1-alpha 1 [EF1A1_MOUSE]	462	60.69	3	1.866E7	52.64	2	1.279E7
Q8VEM8	Phosphate carrier protein, mitochondrial [MPCP_MOUSE]	357	38.36	2	5.860E6	52.43	2	7.930E6
O55143	Sarcoplasmic/endoplasmic reticulum calcium ATPase 2 [AT2A2_MOUSE]	1044	33.29	3	3.871E6	50.45	4	5.011E6
Q60931	Voltage-dependent anion-selective channel protein 3 [VDAC3_MOUSE]	283	51.73	2	6.421E6	50.28	1	8.574E6
O88569	Heterogeneous nuclear ribonucleoproteins A2/B1 [ROA2_MOUSE]	353	35.86	1	2.741E6	49.27	4	3.748E6
Q0GGX2	Zinc finger protein 541 [ZN541_MOUSE]	1363	127.32	8	1.319E7	48.78	2	6.079E6
Q60930	Voltage-dependent anion-selective channel protein 2 [VDAC2_MOUSE]	295	42.06	4	1.245E7	48.37	3	1.123E7
Q6PDQ2	Chromodomain-helicase-DNA-binding protein 4 [CHD4_MOUSE]	1915	38.34	2	2.817E6	47.69	3	2.955E6
Q9D1D4	Transmembrane emp24 domain-containing protein 10 [TMEDA_MOUSE]	219	34.42	1	0.000E0	47.15	3	3.238E6
Q9JMB7	Piwi-like protein 1 [PIWL1_MOUSE]	862	31.88	1	8.271E5	46.26	4	2.922E6
Q501J6	Probable ATP-dependent RNA helicase DDX17 [DDX17_MOUSE]	650	28.16	1	3.101E6	44.72	3	2.649E6
Q921M3	Splicing factor 3B subunit 3 [SF3B3_MOUSE]	1217	28.20	1	1.762E6	44.40	2	2.322E6
Q922R8	Protein disulfide-isomerase A6 [PDIA6_MOUSE]	440	34.98	1	1.568E6	42.26	2	2.322E6
Q6P5H2	Nestin[NEST_MOUSE]	1864	48.87	4	4.569E8	41.67	3	1.485E8
Q9Z2X1	Heterogeneous nuclear ribonucleoprotein F [HNRPF_MOUSE]	415	75.80	3	2.795E6	41.33	2	2.469E6
P67778	Prohibitin [PHB_MOUSE]	272	34.98	2	1.200E6	40.50	2	1.477E6
P00342	L-lactate dehydrogenase C chain [LDHC_MOUSE]	332	61.52	3	3.909E6	40.11	2	1.116E7
Q9DBG6	Dolichyl-diphosphooligosaccharide--protein glycosyltransferase subunit 2 [RPN2_MOUSE]	631	25.71	1	4.215E6	39.28	2	7.582E6
Q80X56	E3 ubiquitin-protein ligase TRIM69 [TRI69_MOUSE]	500	58.73	2	2.273E6	39.22	1	4.108E6
E9Q7E2	AT-rich interactive domain-containing protein 2 [ARID2_MOUSE]	1828	30.10	2	3.943E6	39.08	5	4.280E6
O35737	Heterogeneous nuclear ribonucleoprotein H [HNRH1_MOUSE]	449	103.28	3	3.429E6	38.38	2	3.179E6
P01942	Hemoglobin subunit alpha [HBA_MOUSE]	142	27.35	2	4.142E6	36.53	1	4.191E6
Q9Z1Q9	Valine--tRNA ligase [SYVC_MOUSE]	1263	61.71	4	1.597E6	36.46	2	1.771E6
E9PYK3	Protein mono-ADP-ribosyltransferase PARP4 [PARP4_MOUSE]	1969	131.03	9	9.979E6	36.17	1	2.839E6
P61979	Heterogeneous nuclear ribonucleoprotein K [HNRPK_MOUSE]	463	26.48	1	1.618E6	35.93	2	3.170E6
Q562E2	BTB/POZ domain-containing protein KCTD19 [KCD19_MOUSE]	927	72.33	8	4.212E6	34.09	2	6.260E6
Q9CZ13	Cytochrome b-c1 complex subunit 1, mitochondrial [QCR1_MOUSE]	480	34.85	2	5.214E6	34.07	2	5.291E6
A2A8L1	Chromodomain-helicase-DNA-binding protein 5 [CHD5_MOUSE]	1946	38.34	2	2.817E6	33.63	2	4.662E6
Q8BFZ9	Erlin-2 [ERLN2_MOUSE]	340	37.09	2	2.016E6	33.60	3	2.265E6
Q91Z31	Polypyrimidine tract-binding protein 2 [PTBP2_MOUSE]	531	24.81	2	3.801E6	32.71	1	2.418E6
P01029	Complement C4-B [CO4B_MOUSE]	1738	21.50	2	4.109E6	31.08	1	9.342E6
Q99KI0	Aconitate hydratase, mitochondrial [ACON_MOUSE]	780	43.86	2	2.998E6	30.51	1	4.221E6
Q8BMF4	Dihydrolipoyllysine-residue acetyltransferase component of pyruvate dehydrogenase complex, mitochondrial	642	43.70	4	5.157E6	29.63	2	2.550E6
P10853	Histone H2B type 1-F/J/L [H2B1F_MOUSE]	126	35.81	2	2.211E6	29.44	1	6.213E6
Q91ZW3	SWI/SNF-related matrix-associated actin-dependent regulator of chromatin subfamily A member 5	1051	41.72	3	3.472E6	28.24	1	5.648E6
P97496	SWI/SNF complex subunit SMARCC1 [SMRC1_MOUSE]	1104	31.93	2	1.165E6	22.46	2	1.536E6
P17225	Polypyrimidine tract-binding protein 1 [PTBP1_MOUSE]	555	31.94	2	2.602E6	14.81	2	2.813E6

b

Accession	Description	# AAs	HSF5-C IP (High salt)		
			Score	PSMs	Area
Q5ND04	Heat shock factor protein 5 [HSF5_MOUSE]	624	1772.34	122	7.499E8
Q8K310	Matrin-3 OS=Mus musculus [MATR3_MOUSE]	846	340.79	19	3.785E7
P38647	Stress-70 protein [GRP75_MOUSE]	679	161.58	6	1.091E7
Q501J6	Probable ATP-dependent RNA helicase DDX17 [DDX17_MOUSE]	650	145.70	6	9.757E6
Q8VEK3	Heterogeneous nuclear ribonucleoprotein U [HNRPU_MOUSE]	800	110.11	4	2.389E7
Q9WTM5	RuvB-like 2 [RUVB2_MOUSE]	463	95.34	3	1.161E7
Q01320	DNA topoisomerase 2-alpha [TOP2A_MOUSE]	1528	88.84	5	1.503E7
Q8C2Q3	RNA-binding protein 14 [RBM14_MOUSE]	669	71.11	3	1.125E7
P29341	Polyadenylate-binding protein 1 [PABP1_MOUSE]	636	58.12	2	1.526E7
Q8BL97	Serine/arginine-rich splicing factor 7 [SRSF7_MOUSE]	267	55.37	2	1.317E7
O54941	SWI/SNF-related matrix-associated actin-dependent regulator of chromatin subfamily E member 1	411	54.56	2	6.770E6
P62806	Histone H4 [H4_MOUSE]	103	52.87	4	8.472E6
Q3TWW8	Serine/arginine-rich splicing factor 6 [SRSF6_MOUSE]	339	46.24	2	3.030E6
Q91ZW3	SWI/SNF-related matrix-associated actin-dependent regulator of chromatin subfamily A member 5	1051	44.64	4	7.137E6
Q6PDG5	SWI/SNF complex subunit SMARCC2 [SMRC2_MOUSE]	1213	41.73	4	9.360E6
Q9Z2X1	Heterogeneous nuclear ribonucleoprotein F [HNRPF_MOUSE]	415	39.05	2	4.781E6
P14733	Lamin-B1 [LMNB1_MOUSE]	588	36.78	2	1.347E7
Q9Z204	Heterogeneous nuclear ribonucleoproteins C1/C2 [HNRPC_MOUSE]	313	34.46	2	3.880E6
Q562E2	BTB/POZ domain-containing protein KCTD19 [KCD19_MOUSE]	927	30.34	2	4.025E6
Q60749	KH domain-containing, RNA-binding, signal transduction-associated protein 1 [KHDR1_MOUSE]	443	24.09	3	5.713E6

c

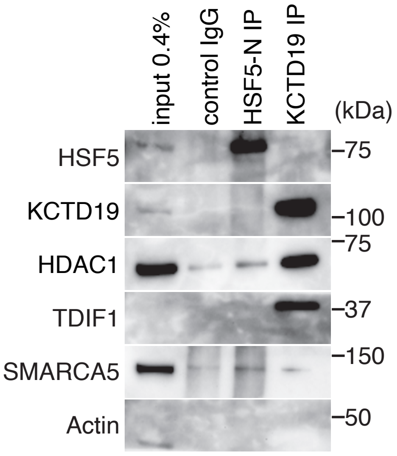

Supplementary Figure 9. MS analyses of HSF5 interacting factors in testis extracts.

(a) The immunoprecipitates (IP) by two different HSF5-N antibodies (HSF5-N1, HSF5-N2) from the chromatin-bound fractions of the testis extracts were subjected to liquid chromatography tandem-mass spectrometry (LC-MS/MS) analyses. The proteins identified by the LC-MS/MS analysis of HSF5N-IP are presented after excluding the proteins detected in the control IgG-IP. The proteins that were detected in both HSF5-N1 IP and HSF5-N2 IP with more than 1 different peptide hits are listed with SwissProt accession number, Amino acid length, the number of peptide hits and Mascot scores.

(b) The immunoprecipitates (IP) by HSF5-C antibody from the chromatin-bound fractions of the testis extracts. The proteins identified by the LC-MS/MS analysis of HSF5C-IP are presented as in A. See also Supplementary Data 7 for the raw data of LC-MSMS analysis.

Note that SMARCA5 and KCTD19 was repeatedly identified in the immunoprecipitates by HSF5-N1, HSF5-N2 HSF5-C antibodies.

(c) Immunoblot showing the immunoprecipitates of HSF5 from testis chromatin extracts.

Figure 1d

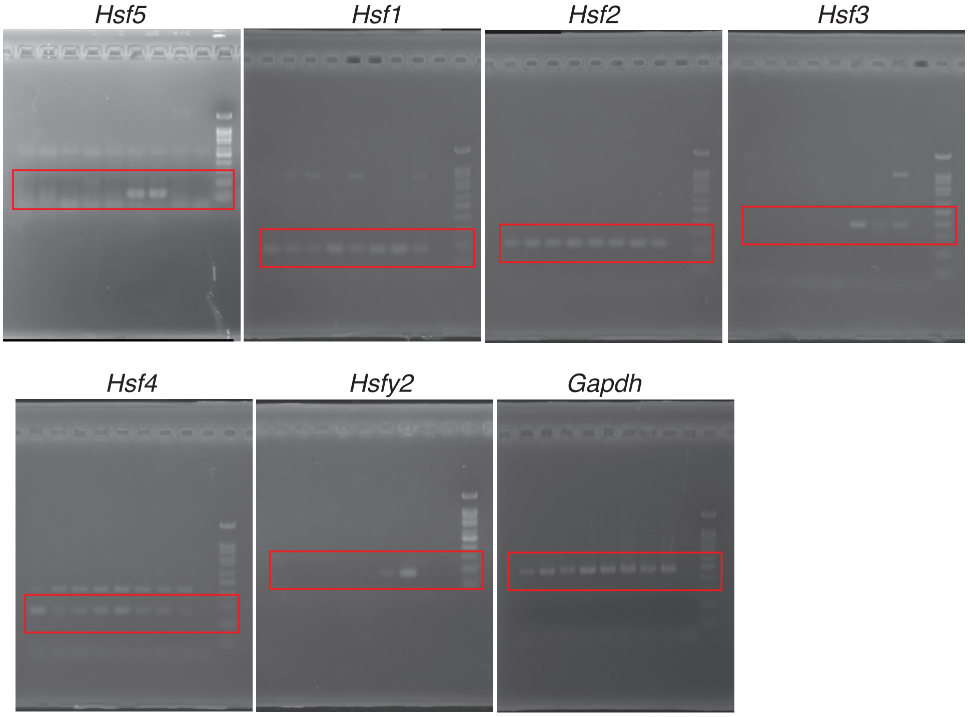

Figure 2c

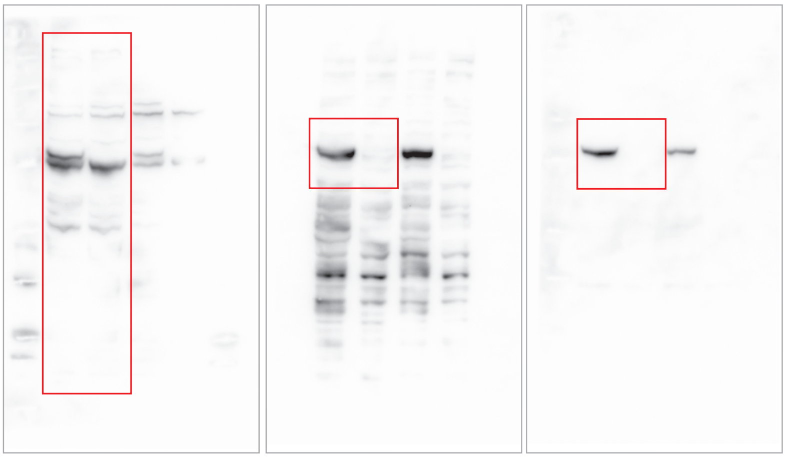

Figure 3b

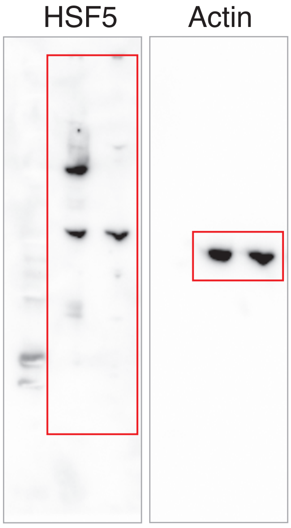

Figure S9c

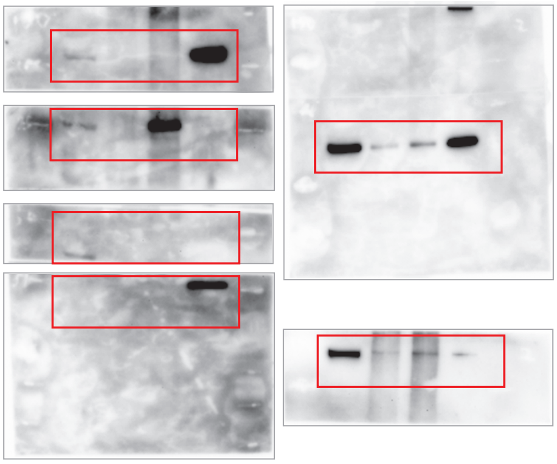

Figure 8b

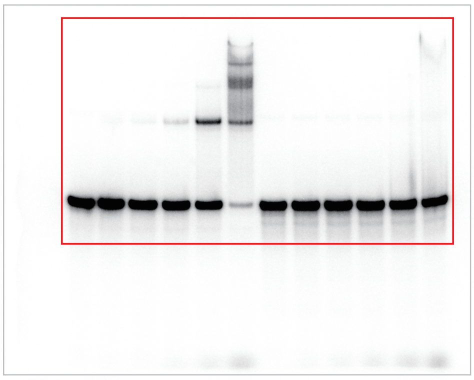

Figure 8c

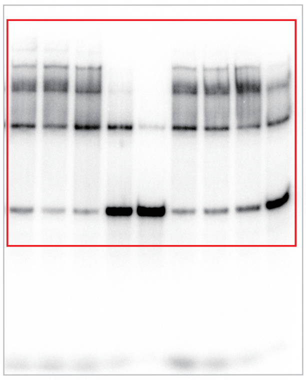

Supplementary Figure 10. Uncropped images of gels and blots.
Full-length / uncropped images of agarose gel (Fig1d), western blot (Fig2c, Fig3b, Fig S9c) and the scanned images of autoradiograph (Fig8b, Fig8c) are shown.
