# Supplementary material for: Atypical heat shock transcription factor HSF5 is critical for male meiotic prophase under non-stress conditions
Source: Nat Commun. 2024 Apr 29;15:3330. doi: 10.1038/s41467-024-47601-0 (PMC11059408; doi:10.1038/s41467-024-47601-0)
Supplement: Supplementary file 3 — Description of Additional Supplementary Files [file 41467_2024_47601_MOESM3_ESM.pdf]

Description of Additional Supplementary Files

File Name: Supplementary Data 1
Description: Primers and oligos used in this study.

File Name: Supplementary Data 2
Description: The list of DEGs in the RNA-seq of the *Hsf5+/-* versus *Hsf5* KO testes.
Tab 1: Differentially expressed genes between 33℃ and 37℃ in *Hsf5* +/- control.
Tab 2: Differentially expressed genes between 33℃ and 37℃ in *Hsf5* KO.
Tab 3: Overlap of the DEGs in 37 ℃ versus 33 ℃ between *Hsf5* +/- and *Hsf5* KO.

File Name: Supplementary Data 3
Description: The list of DEGs in the SMART RNA-seq of the control versus *Hsf5* KO spermatocytes.
Tab 1: The complete list of expression profile of all genes that were detected by SMART RNA-seq.
Tab 2: The complete result of up-regulated genes in *Hsf5* KO spermatocytes.
Tab 3: The complete result of down-regulated genes in *Hsf5* KO spermatocytes.
Tab 4: The complete result of Gene enrichment analysis for the up-regulated genes in *Hsf5* KO spermatocytes.
Tab 5: The complete result of Gene enrichment analysis for the down-regulated genes in *Hsf5* KO spermatocytes.
Tab 6: The down-regulated genes in *Hsf5* KO that are shown in the heatmap.
Tab 7-11: The complete result of Gene enrichment analysis for the down-regulated genes in *Hsf5* KO spermatocytes in the clusters 1- 5.

File Name: Supplementary Data 4
Description: The list of DEGs in the clusters of scRNA-seq of testicular germ cells.
Tab 1: Shown are the complete list of DEGs in the clusters of scRNA-seq at P16.
Tab 2-13: The complete result of Gene enrichment analysis for the highly expressed genes in the clusters are shown in other tabs (C0-C11).
Tab 14: Differentially expressed genes between WT and *Hsf5* KO of clusters 2, 3, and 9.
Tab 15: Downregulated genes in *Hsf5* KO of clusters 2, 3, and 9.
Tab 16: Downregulated genes in *Hsf5* KO (SMART-seq)
Tab 17: Overlap of the downregulated genes in *Hsf5* KO between scRNA-seq and SMART-seq

File Name: Supplementary Data 5
Description: The gene list of HSF5-ChIP targets

Tab 1: The complete result of HSF5-ChIP targets.
Tab 2: The complete result of Gene enrichment analysis for the HSF5-ChIP target genes.
Tab 3: The HSF5-bound genes (Class1-7) that are shown in the heat map of the hierarchical clustering.
Tab 4: The HSF1- and HSF2- bound genes in the testis are shown.
Tab 5: The HSF1- and HSF2- bound gene sets that were described in the originally reported papers in previous ChIP on Chip studies (Akerfelt et al. 2008), (Akerfelt et al. 2010).

File Name: Supplementary Data 6
Description: The gene list of HSF5-CUT&Tag targets
Tab 1: The complete result of CUT&Tag target sites commonly identified by different HSF5 antibodies.
Tab 2: The complete result of CUT&Tag target genes commonly identified by different HSF5 antibodies.
Tab 3: The HSF5-bound genes (Class1-3) that are shown in the heat map of the hierarchical clustering.
Tab 4: The complete result of Gene enrichment analysis for Class 1 HSF5-CUT&Tag target genes.
Tab 5: The complete result of Gene enrichment analysis for Class 2 HSF5-CUT&Tag target genes.
Tab 6: The complete result of Gene enrichment analysis for Class 3 HSF5-CUT&Tag target genes.
Tab 7: The complete result of ChIP-seq target sites commonly identified by different antibodies.
Tab 8: The commonly identified sites in both ChIP-seq and CUT&Tag.
Tab 9: Gene list comparison between HSF5 CUT&Tag and HSF1 target genes.
Tab 10: Gene list comparison between HSF5 CUT&Tag and HSF2 target genes.
Tab 11: Gene list comparison between HSF5 CUT&Tag and HSF1, HSF2 target genes.

File Name: Supplementary Data 7
Description: The complete result of HSF5 IP-MS results
Tab 1: The complete list of HSF5-N1 and HSF5-N2 IP proteins from chromatin-bound fractions of the testis extracts identified by LC-MSMS.
Tab 2: The complete list of HSF5-N1 and HSF5-N2 IP proteins identified from cytosol fractions of the testis extracts by LC-MSMS.
Tab 3: The complete list of HSF5-C IP proteins identified from chromatin-bound fractions and cytosol fractions of the testis extracts by LC-MSMS.
Raw data are shown in other tabs.
