# Supplementary material for: Atypical heat shock transcription factor HSF5 is critical for male meiotic prophase under non-stress conditions
Source: Nat Commun. 2024 Apr 29;15:3330. doi: 10.1038/s41467-024-47601-0 (PMC11059408; doi:10.1038/s41467-024-47601-0)
Supplement: Supplementary file 11 — Reporting Summary [file 41467_2024_47601_MOESM11_ESM.pdf]

nature research

Corresponding author(s): Kei-ichiro Ishiguro

Last updated by author(s): Apr 2, 2024

Reporting Summary

Nature Research wishes to improve the reproducibility of the work that we publish. This form provides structure for consistency and transparency in reporting. For further information on Nature Research policies, see our Editorial Policies and the Editorial Policy Checklist.

Statistics

For all statistical analyses, confirm that the following items are present in the figure legend, table legend, main text, or Methods section.

n/a	Confirmed	
☐	☒	The exact sample size (n) for each experimental group/condition, given as a discrete number and unit of measurement
☐	☒	A statement on whether measurements were taken from distinct samples or whether the same sample was measured repeatedly
☐	☒	The statistical test(s) used AND whether they are one- or two-sided<br>*Only common tests should be described solely by name; describe more complex techniques in the Methods section.*
☒	☐	A description of all covariates tested
☐	☒	A description of any assumptions or corrections, such as tests of normality and adjustment for multiple comparisons
☐	☒	A full description of the statistical parameters including central tendency (e.g. means) or other basic estimates (e.g. regression coefficient) AND variation (e.g. standard deviation) or associated estimates of uncertainty (e.g. confidence intervals)
☐	☒	For null hypothesis testing, the test statistic (e.g. F, t, r) with confidence intervals, effect sizes, degrees of freedom and P value noted<br>*Give P values as exact values whenever suitable.*
☒	☐	For Bayesian analysis, information on the choice of priors and Markov chain Monte Carlo settings
☒	☐	For hierarchical and complex designs, identification of the appropriate level for tests and full reporting of outcomes
☒	☐	Estimates of effect sizes (e.g. Cohen's d, Pearson's r), indicating how they were calculated

Our web collection on statistics for biologists contains articles on many of the points above.

Software and code

Policy information about availability of computer code

Data collection	no software was used for the data collection.
Data analysis	R (version 4.2.2), Microsoft Excel (version 16.48), SoftWoRx (ver.7.2.1, GE Healthcare)., Seurat packge for R (v.3.1.3), monocle (ver.2.14.0), RStudio (ver.2021.9.2.382), Bioworks (Ver. 3.3; Thermo Scientific), Xcalibur (Version 4.0, Thermo Fisher Scientific)

For manuscripts utilizing custom algorithms or software that are central to the research but not yet described in published literature, software must be made available to editors and reviewers. We strongly encourage code deposition in a community repository (e.g. GitHub). See the Nature Research guidelines for submitting code & software for further information.

Data

Policy information about availability of data

All manuscripts must include a data availability statement. This statement should provide the following information, where applicable:

- Accession codes, unique identifiers, or web links for publicly available datasets
- A list of figures that have associated raw data
- A description of any restrictions on data availability

All data supporting the conclusions are present in the paper and the supplementary materials. A reporting summary for this Article is available as Supplementary Information file. The source data (for Fig. 1c, Fig. 1e, Fig. 3d, Fig. 3h, Fig. 4a, Fig. 4b, Fig. 4e, Fig. 4f, Fig. 4g, Fig. 5a, Fig. 5d, Fig. 6c, Fig. 6j, Fig. 6k, Fig. 7e, Fig. S1a, Fig. S3b, Fig. S3c, Fig. S4c) are provided with this paper. Sequencing data have been deposited in DDBJ Sequence Read Archive (DRA) under the accession DRA 017033 for scRNA-seq data of P16 WT and Hsf5 knockout testes, DRA017058 (DRR502790, DRR502791) for the SMART RNA-seq data of the P16 sorted meiotic prophase spermatocytes, DRA017059 (DRR502792-DRR502794) for ChIP-seq data of WT testes, PRJDB16509 (DRR530443- DRR530448) for CUT&Tag data of WT, Hsf5 +/- and Hsf5 KO spermatocytes, PRJDB16509 (DRR530449-DRR530460) for RNA-seq of whole testes of WT and knockout mice at 33 degree C and 37degree C, DRR540205, DRR540206 for ATAC-seq data of the control and Hsf5 KO spermatocytes, respectively.

Mass spectral data of HSF5 IP have been deposited in Japan ProteOme STandard Repository (jPOSTrepo) under the accession PXD051085 for ProteomeXchange and JPST003018 for jPOST [https://repository.jpostdb.org/entry/JPST003018].
MEIOSIN ChIP-seq data was derived from DDBJ DRA007778. The scRNA-seq data of mouse adult testis was derived from GEO: GSE109033.
Uncropped blots can be found in Supplementary Fig S10.
Reference genome for scRNA-seq, mm10 is obtained from following URL: https://support.10xgenomics.com/single-cell-gene-expression/software/release-notes/build#mm10_3.0.0
Original images and can be obtain from Figshare: https://doi.org/10.6084/m9.figshare.24160956

Field-specific reporting

Please select the one below that is the best fit for your research. If you are not sure, read the appropriate sections before making your selection.

- [x] Life sciences
- [ ] Behavioural & social sciences
- [ ] Ecological, evolutionary & environmental sciences

For a reference copy of the document with all sections, see nature.com/documents/nr-reporting-summary-flat.pdf

Life sciences study design

All studies must disclose on these points even when the disclosure is negative.

Sample size	No statistical method was used to predetermine sample size. We followed the conventional way of quantification accepted in many of the published paper in meiosis research field and determined the sample size according to published papers (Horisawa-Takada, Y. et al. (2021) doi:https://doi.org/10.1038/s41467-021-23378-4, Ishiguro, K. I. et al. (2020) doi:10.1016/j.devcel.2020.01.010, Larose, H. et al., (2020) doi:10.1091/mbc.E20-05-0334.).
Data exclusions	No data was excluded.
Replication	Each conclusion in the manuscript was based on results that were reproduced in at least two independent experiments and in at least two or three independent mice of each genotype, unless otherwise stated.
Randomization	Mice were categorized based on their genotypes. The genotypes were determined by PCR. For experiments other than those involving mice, samples were non-randomly chosen according to the genotype.
Blinding	The investigators were not blinded to allocation during the experiments or to outcome assessment.<br>This is because the phenotypes were quite obvious that observer can be sure without blind test.<br>Further, the observer unbiasedly and carefully performed the quantification with enough sample number to make sure the conclusion.

Reporting for specific materials, systems and methods

We require information from authors about some types of materials, experimental systems and methods used in many studies. Here, indicate whether each material, system or method listed is relevant to your study. If you are not sure if a list item applies to your research, read the appropriate section before selecting a response.

Materials & experimental systems

n/a	Involved in the study	
[ ]	[x]	Antibodies
[x]	[ ]	Eukaryotic cell lines
[x]	[ ]	Palaeontology and archaeology
[ ]	[x]	Animals and other organisms
[x]	[ ]	Human research participants
[x]	[ ]	Clinical data
[x]	[ ]	Dual use research of concern

Methods

n/a	Involved in the study	
[ ]	[x]	ChIP-seq
[x]	[ ]	Flow cytometry
[x]	[ ]	MRI-based neuroimaging

Antibodies

Antibodies used	The following antibodies were used for immunoblot (IB) and immunofluorescence (IF) studies: Guinea pig anti-SYCP3 (IF, 1:2000, our home made) (Ishiguro et al. 2020), Rat anti-SYCP3 (IF, 1:1000, our home made) (Ishiguro et al. 2020), Rabbit anti-SYCP1 (IF, 1:1000, Abcam ab15090), Mouse anti-γH2AX (IF, 1:1000, Abcam ab26350), Mouse anti-γH2AX (IF, 1:1000, Merck Millipore 05-636), Rabbit anti-γH2AX (IF, 1:1000, Abcam ab11174), Rat anti-STRA8 (IF, 1:1000, our homemade) (Ishiguro et al. 2020), Guinea pig anti-H1t (IF, 1:2000, kindly provided by Marry Ann Handel)(Cobb et al. 1999), Rabbit anti-α-tubulin DM1A (IB, 1:2000, Sigma 05-829), Rabbit anti-HSF5 N (IB,IF, 1:1000, our home made)(this paper), Rabbit anti-HSF5 C (IB,IF, 1:1000, our home made)(this paper), Mouse anti-FLAG M2 (IB, 1:1000, Sigma-Aldrich F1804), Rabbit anti-HA (IB,IF, 1:1000, Abcam ab9110), Mouse anti-MLH1 (IF, 1:200, Cell Signaling 3515), Rabbit anti-DMC1 (IF, 1:500, Santa Cruz SC-22768), Mouse anti-RNA Pol II (8WG16) (IF, 1:200, Santa Cruz : SC-56767), Rabbit

anti-BRCA1 (IF, 1:2000, kindly provided by Satoshi Namekawa) (Tanno et al. 2022), Rabbit anti-KCTD19-N (IB, 1:1000, our home made)(Horisawa-Takada et al. 2021), Rabbit anti-HDAC1(IB, 1:1000, Abcam ab19845), Rabbit anti-TDIF1(IB, 1:1000, Abcam ab228703), Rabbit anti-SNF2H/SMARCA5 (IF, 1:1000, Abcam ab72499) , Rabbit anti-Actin (IB, 1:1000, Sigma A2066).

Validation

The newly generated antibodies in this study were validated by western bloting and immunostaining.
The following our home made antibodies were validated for immunofluorescence (IF) in our previous studies: Guinea pig anti-SYCP3 (IF, 1:2000, our home made) (Ishiguro et al. 2020), Rat anti-SYCP3 (IF, 1:1000, our home made) (Ishiguro et al. 2020) , Rat anti-STRA8 (IF, 1:1000, our homemade) (Ishiguro et al. 2020), Rabbit anti-KCTD19-N (IB, 1:1000, our home made)(Horisawa-Takada et al. 2021).
The following antibodies were validated for immunoblot (IB) and immunofluorescence (IF) in manufacture's website :
Rabbit anti-SYCP1 (IF, 1:1000, Abcam ab15090), Mouse anti-γH2AX (IF, 1:1000, Abcam ab26350), Mouse anti-γH2AX (IF, 1:1000, Merck Millipore 05-636), Rabbit anti-γH2AX (IF, 1:1000, Abcam ab11174), Guinea pig anti-H1t (IF, 1:2000, kindly provided by Marry Ann Handel)(Cobb et al. 1999), Rabbit anti-α-tubulin DM1A (IB, 1:2000, Sigma 05-829), Mouse anti-FLAG M2 (IB, 1:1000, Sigma-Aldrich F1804), Rabbit anti-HA (IB,IF, 1:1000, Abcam ab9110), Mouse anti-MLH1 (IF, 1:200, Cell Signaling 3515), Rabbit anti-DMC1 (IF, 1:500, Santa Cruz SC-22768), Mouse anti-RNA Pol II (8WG16) (IF, 1:200, Santa Cruz : SC-56767), Rabbit anti-BRCA1 (IF, 1:2000, kindly provided by Satoshi Namekawa) (Tanno et al. 2022), Rabbit anti-HDAC1(IB, 1:1000, Abcam ab19845), Rabbit anti-TDIF1(IB, 1:1000, Abcam ab228703), Rabbit anti-SNF2H/SMARCA5 (IF, 1:1000, Abcam ab72499) , Rabbit anti-Actin (IB, 1:1000, Sigma A2066).

Animals and other organisms

Policy information about studies involving animals; ARRIVE guidelines recommended for reporting animal research

Laboratory animals

Hsf5 knockout (Hsf5 KO) mice were with the C57BL/6 background. Hsf5-3xFLAG-HA knock-in mice were C57BL/6 background (age : 5-weeks old). Male mice were used for ChIP-seq (age : postnatal day 10-21 old), Cut&Tag (age : postnatal day 18-19 old), immunoprecipitation of testis extracts (age : postnatal day 18-23 old, 4-weeks and 8-weeks old), histological analysis of testes, and immunostaining of testes, RNA extraction (age : postnatal day 15-21, 4-weeks and 8-weeks old). Female mice were used for histological analysis of the ovaries and RNA extraction (age : postnatal 4-weeks and 8-weeks old) (embryonic day 12.5-18.5).
Housing conditions for the mice were under 12 hours dark/12 hours light cycle, ambient temperature at 20-23 degree C and humidity 40-60 % .
Antibody production using rabbit was done by a contractor (Kiwa Laboratory Animals Co., Ltd.). The handling of these animals was carried out according the contractor's animal experimental protocols.

Wild animals

No wild animal was used.

Field-collected samples

No field-collected samples were used in the study.

Ethics oversight

Animal experiments were approved by the Institutional Animal Ethics Committees of Kumamoto University (approval F28-078, A 2022-01).

Note that full information on the approval of the study protocol must also be provided in the manuscript.

ChIP-seq

Data deposition

- [x] Confirm that both raw and final processed data have been deposited in a public database such as GEO.
- [x] Confirm that you have deposited or provided access to graph files (e.g. BED files) for the called peaks.

Data access links
May remain private before publication.

The HSF5 ChIP-seq data of mouse testes are deposited in the DDBJ Sequence Read Archive under the accession DRA017059. The HSF5 Cut&Tag data are deposited in the DDBJ Sequence Read Archive under the accession DRA. Processed data files can be obtain from Figshare: https://doi.org/10.6084/m9.figshare.24160956.

Files in database submission

PROCESSED DATA FILES
ChIP_Input.BigWig
ChIP_ControlIgG.BigWig
ChIP_HSF5-C.BigWig
CT_ControlIgG_norm.BigWig
CT_HSF5-N1_norm.BigWig
CT_HSF5-N2_norm.BigWIg
CT_HSF5-C_norm.BigWig
CT_Het_Control_IgG_norm.BigWig
CT_Het_HSF5-C_norm.BigWig

raw files (SRA)
ChIP_Control IgG: DRR502793
ChIP_input: DRR502792
ChIP HSF5-C: DRR502794
CUT&Tag Control IgG: DRR530445
CUT&Tag HSF5-N1: DRR530446
CUT&Tag HSF5-N2: DRR530447
CUT&Tag HSF5-C: DRR530448

	CUT&Tag Het Control IgG: DRR530443<br>CUT&Tag Het HSF5-C: DRR530444
Genome browser session<br>(e.g. UCSC)	UCSC mm10

Methodology

Replicates	Cut&Tag were performed with three different anti-HSF5-N1, -N2, -C antibodies. ChIP-seq were performed with anti-HSF5 -C.
Sequencing depth	ChIP_Control IgG: DRR502793; 32,029,487-76bp, single-end<br>ChIP_input: DRR502792; 35,840,250-76bp, single-end<br>ChIP HSF5-C: DRR502794; 35,393,362-76bp, single-end<br>CUT&Tag Control IgG: DRR530445; 29,378,807-76bp, single-end<br>CUT&Tag HSF5-N1: DRR530446; 2,508,095-76bp, single-end<br>CUT&Tag HSF5-N2: DRR530447; 26,209,116-76bp, single-end<br>CUT&Tag HSF5-C: DRR530448; 30,736,775-76bp, single-end<br>CUT&Tag Het Control IgG: DRR530443; 25,801,764-76bp, single-end<br>CUT&Tag Het HSF5-C: DRR530444; 22,150,182-76bp, single-end
Antibodies	rabbit anti-HSF5-C, rabbit anti-HSF5-N1, rabbit anti-HSF5-N2
Peak calling parameters	ChIP Peak calling was performed using MACS program v2.1.0 (Zhang et al., 2008) (https://github.com/macs3-project/MACS) with the option (-g mm -p 0.00001).<br>Cut&Tag peak calling was performed using control IgG data as control using SEACR ver. 1.3. Using bedtools, common peaks among HSF5-N1, N2 and C antibodies but not control IgG were obtained as HSF5-bound site.
Data quality	FastQC (version 0.11.17) was used for initial quality control of the reads.<br>ChIP_Control IgG: DRR502793; 2 peaks<br>ChIP HSF5-C: DRR502794; 165peaks<br>CUT&Tag HSF5-N1: DRR530446, 2,514 peaks<br>CUT&Tag HSF5-N2: DRR530447, 3,685 peaks<br>CUT&Tag HSF5-C: DRR530448, 3,633 peaks<br>CUT&Tag Het HSF5-C: DRR530444, 6,810 peaks
Software	Bowtie2 v2.3.4.1, MACS program v2.1.0 (Zhang et al., 2008) (https://github.com/macs3-project/MACS) for Peak calling, bedtools program (v2.27.1) (Quinlan and Hall, 2010) (https://bedtools.readthedocs.io/en/latest/)., Cis-regulatory Element System (CEAS) v0.9.9.7 (package version 1.0.2), MEME-ChIP v5.1.1 website (http://meme-suite.org/tools/meme-chip) (Bailey, 2011) for Motif identification, deepTools (v3.1.0) for generation of BigWig files, Integrative Genomics Viewer software (v.2.8.3) http://software.broadinstitute.org/software/igv/home. , DAVID Bioinformatics Resources 6.8 (Huang da et al., 2009) (https://david.ncifcrf.gov/), deeptools program v3.5.0. for aggregation plots and heatmaps, GREAT website (v4.0.4) (http://great.stanford.edu/public/html/), bedtools program (v2.27.1)
